# The suppression of MAPK/NOX/MMP signaling prompts renoprotection conferred by prenatal naproxen in weaning preeclamptic rats

**DOI:** 10.1038/s41598-023-44617-2

**Published:** 2023-10-15

**Authors:** Sherien A. Abdelhady, Mennatallah A. Ali, Dalia M. Yacout, Marwa M. Essawy, Lamia S. Kandil, Mahmoud M. El-Mas

**Affiliations:** 1 Department of Pharmacology and Therapeutics, Faculty of Pharmacy, Pharos University in Alexandria, Canal El Mahmoudia Street, Alexandria, 21568 Egypt; 2 Department of Clinical Pharmacology, Faculty of Medicine, Alexandria University, Alexandria, Egypt; 3 Department of Oral Pathology, Faculty of Dentistry, Alexandria University, Alexandria, Egypt; 4 Center of Excellence for Research in Regenerative Medicine and Applications (CERRMA), Faculty of Medicine, Alexandria University, Alexandria, Egypt; 5 School of Pharmacy and Biomedical Sciences, University of Central Lancashire, Preston, UK; 6 Department of Pharmacology and Toxicology, Faculty of Pharmacy, Alexandria University, Alexandria, Egypt; 7 Department of Pharmacology and Toxicology, College of Medicine, Kuwait University, Kuwait City, Kuwait

**Keywords:** Disease model, Reprogramming, Pharmacology, Nephrology, Biomarkers

## Abstract

Although nonsteroidal antiinflammatory drugs (NSAIDs) are frequently used for fever and pain during pregnancy, their possible interaction with perinatal renal injury induced by preeclampsia (PE) has not been addressed. Here, studies were undertaken in the *N*(gamma)-nitro-l-arginine methyl ester (l-NAME) PE model to assess the influence of gestational NSAIDs on renal damage in weaning dams. PE-evoked increments and decrements in urine protein and creatinine clearance, respectively, were intensified by celecoxib and weakened by diclofenac or naproxen. Naproxen also improved renal cloudy swelling, necrosis, and reduced glomerular area evoked by PE. The concomitant rises in renal expression of markers of oxidative stress (NOX2/4), extracellular matrix metaloproteinase deposition (MMP9), and prostanoids (PGE_2_, PGF2α, TXA2) were all more effectively reduced by naproxen compared with celecoxib or diclofenac. Western blotting showed tripled expression of mitogen-activated protein kinases (MAPKs; p-p38, p-JNK1, p-ERK1, p-ERK2) in PE kidneys that was overturned by all NSAIDs, with naproxen producing the largest drop in p-ERK2 expression. The PE-provoked elevation in renal expression of autophagic marker LC3 was reduced by naproxen and diclofenac, but not celecoxib. The data suggests superior effect for naproxen over other NSAIDs in rectifying preeclamptic renal injury and predisposing inflammatory, oxidative, autophagic, and fibrotic signals.

## Introduction

Preeclampsia (PE) is a pregnancy-related syndrome characterized by a widespread endothelial dysfunction and vasospasm that negatively influences remote organs, including the kidney. The pathogenic trails of PE begin with defective placental cytotrophoblast invasion and spiral arteries remodeling that progresses to placental hypoxia and consequent unfavorable milieus of PE^1–3^. Proinflammatory cytokines^4^, mitogen-activated protein kinases (MAPKs)^5^, reactive oxygen species, matrix metalloproteinases (MMPs)^6^, apoptotic enzymes, and autophagosomes^7^ are offending pathways that critically contribute to PE onset and progression. The renal injurious action of PE is associated with proteinuria, glomerular endotheliosis, and tubular and vascular damage^8,9^. The unraveling of the bidirectional rapport of PE and kidney diseases is puzzling. Renal dysfunction caused by PE has been accounted for by directly damaging the glycocalyx and podocytes^10^ or indirectly via increasing the incidence of hypertension, diabetes mellitus, and dyslipidemia^11,12^.

Disturbances in cyclooxygenase (COX) pathway and arachidonate end products have been implicated in the pathophysiology and complications of PE. For instance, imbalances in prostacyclin and thromboxane, two COX metabolites of arachidonic acid, account for principal clinical symptoms associated with PE like hypertension, platelet aggregation, and compromised uteroplacental blood flow^13^. COX-1 and COX-2 have also been shown to contribute to the development of angiogenesis inhibitor-induced preeclamptic manifestation of hypertension and renal injury^14,15^. COX inhibition by aspirin rectifies the preeclamptic prostacyclin/thromboxane ratio^16^, blunt oxidative stress^17^, and reduce the incidence of PE in high-risk patients^17^. Furthermore, COX inhibition effectively improves preeclamptic myocardial damage, but had no effect or even exaggerated the concomitant rise in blood pressure^18^. Case reports showed that indomethacin exacerbates the PE-evoked hypertension^19,20^ and weaken the antihypertensive efficacy of β-adrenergic receptors blockers against preeclamptic hypertension^21^. NSAIDs are often prescribed during pregnancy to treat fever, pain and inflammation^22^. Notably, the COX inhibitory effects and reduced generation of renal vasodilator prostanoids have been implicated in the NSAID-induced nephrotoxicity. The latter is a recognized adverse effect of NSAIDs that manifest as acute or chronic kidney injury, electrolyte and acid–base disorders, and interstitial nephritis^23,24^. Notably, reported findings on the use of NSAIDs during pregnancy are inconsistent. Some authors argued against the use of COX-2 inhibitors during the last semester, but no data was provided to support such a claim^25^. Others failed to demonstrate significant maternal or neonatal adverse events and revealed a safer short-term profile for celecoxib compared with indomethacin^26^.

Despite the reported isolated renal damaging effects of PE^8,9^ and NSAIDs^23^, we are not aware of any study that assessed renal consequences of the exposure to the two interventions simultaneously. This was accomplished in the current investigation by evaluating functional and histopathological indices of renal function in PE rats treated during the last third of pregnancy with naproxen, celecoxib, or diclofenac. The latter compounds have been shown to exhibit variable COX-1/2 selectivity, with celecoxib being a selective COX-2 inhibitor while the other two NSAIDs elicits nonselective COX-1/2 inhibition^27^. Gene and protein expression studies were also undertaken to determine the roles of arachidonate end products, MAPK signaling, and downstream oxidative, fibrotic, and autophagic effectors in the interaction.

## Results

### Effects of gestational NSAIDs on renal function and architecture

**Figure 1:**
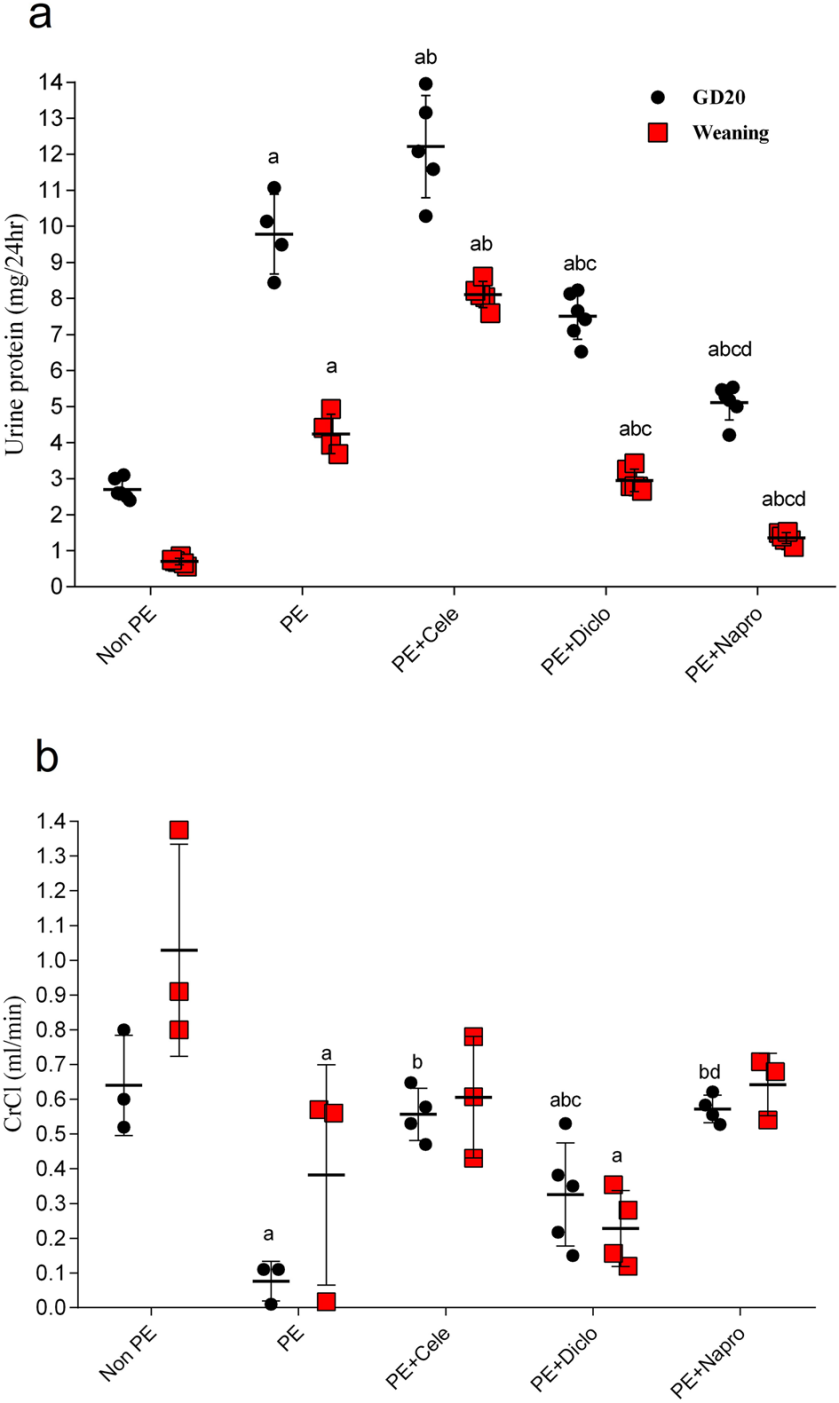
Effect of prenatal administration of celecoxib, diclofenac or naproxen on urine protein (**a**) and creatinine clearance (CrCl, **b**) in PE rats at gestational day 20 (GD20) and weaning time. Values are means ± S.D. of 3–5 rats. *P < 0.05 vs. non-PE group (a), PE (b), PE + celecoxib (c), and PE + diclofenac (d) according to two-way ANOVA (*F*-statistics, *p* > 0.0001).

As depicted in Fig. 1a, the protein contents in the 24-h urine samples collected from PE dams at GD20 and weaning time were significantly higher than respective values seen in control (non-PE) rats. The rises in urine protein evoked by PE were intensified after gestational administration of celecoxib (10 mg/kg) and attenuated by gestational diclofenac (0.5 mg/kg) and naproxen (1 mg/kg). Further, the induction of PE was accompanied by dram/atic and significant decrements in creatinine clearance that were partially alleviated by concurrently administered NSAIDs (Fig. 1b). Naproxen was more effective than celecoxib or diclofenac in restoring the altered urine protein and CrCl back to non-PE levels (Fig. 1).

**Figure 2:**
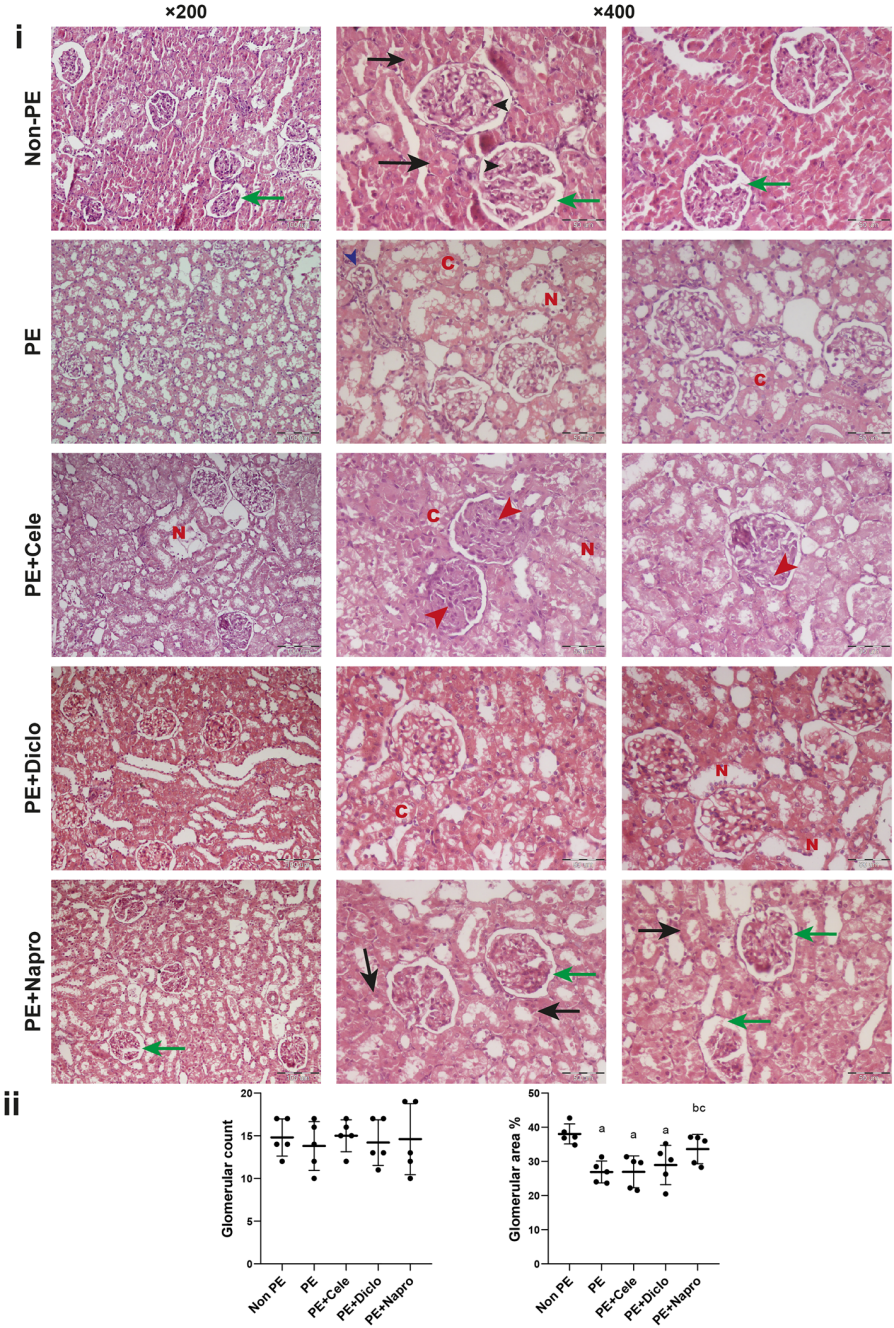
Representative kidney photomicrographs demonstrating the effect of prenatal administration of celecoxib, diclofenac, or naproxen in PE rats. (**i**) H&E-stained renal photomicrographs (×200; scale bar = 100 μm and ×400; scale bar = 50 μm) of non-PE rats showed normal glomeruli cellularity with opened capillary loops (black arrowheads), standard tubular epithelial lining (black arrow) and intact Bowman’s space (green arrows). PE kidneys sjowed signs of glomerular shrinkage (blue arrowhead), cloudy swelling (C), and fatty degeneration of tubular epithelial lining and necrosis (N). PE + cele and PE + Diclo kidneys display similar renal histological alterations to the PE model in terms of narrow mesangial cell proliferation (red arrowheads) with glomerular capillary endotheliosis, in addition to cloudy swelling (C) of the tubular epithelial lining, and necrosis (N). The PE + Napro kidney reveals closer architecture to the kidney of control non-PE rat in terms of intact Bowman’s space (green arrows), normal tubular epithelial lining (black arrows). (**ii**) The scatter plot of the assessed histomorphometric parameters (glomerular number and surface area). Values are means ± S.D. of 4–5 rats. Non-PE group (a), PE (b), PE + celecoxib (c), and PE + diclofenac (d) according to one-way-ANOVA (*F*-statistics, *p* > 0.0001).

The reprogramming effect of gestational NSAIDs on modifications caused by PE in kidney architecture is shown in Fig. 2. The glomeruli in kidneys of PE dams showed mesangial cell proliferation with glomerular capillary endotheliosis. Few of the glomerular tufts displayed shrinkage, atrophy, and necrosis. The tubular epithelial lining showed severe degree of degeneration represented by cloudy swelling intermingled with areas of necrosis (Fig. 2i). Gestational treatment with celecoxib or diclofenac did not improve the distorted histological picture caused by PE. Meanwhile, naproxen treatment effectively preserved the glomerular space and diminished mesangial cell proliferation. Additionally, naproxen reduced the preeclamptic interstitial inflammation and restored the standard architecture of the tubular epithelial lining (Fig. 2i). Morphometrically, kidneys of PE rats showed similar glomerular counts to those of respective non-PE values, but significantly smaller glomerular areas. The reductions in glomerular area induced by PE were reversed by naproxen and not by celecoxib or diclofenac (Fig. 2ii).

### Effect of gestational NSAIDs on renal prostanoids

**Figure 3:**
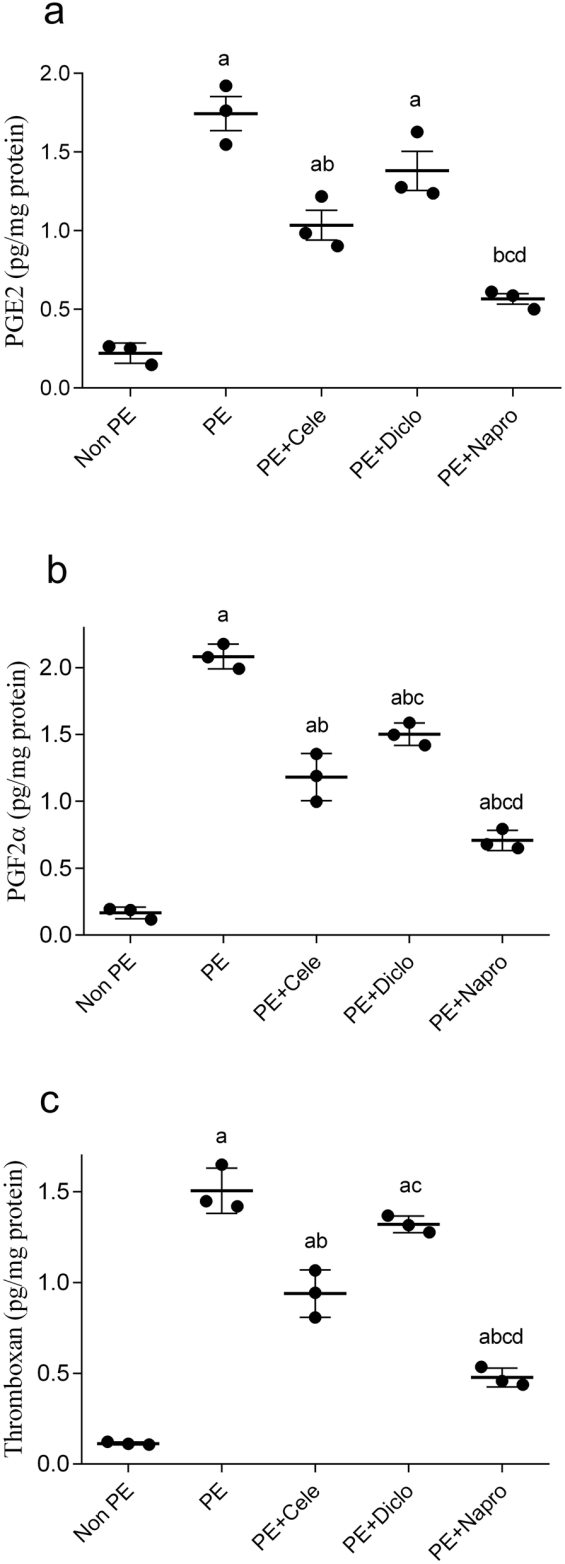
Effect of prenatal administration of celecoxib, diclofenac or naproxen on renal PGE2 (**a**), PGF2α (**b**) and thromboxane A2 (**c**) in weaning PE rats. Values are means ± S.D. of 3 rats. *P < 0.05 vs. non-PE (a), PE (b), PE + celecoxib (c), and PE + diclofenac (d) according to one-way-ANOVA (*F*-statistics, *p* > 0.0001).

**Figure 4:**
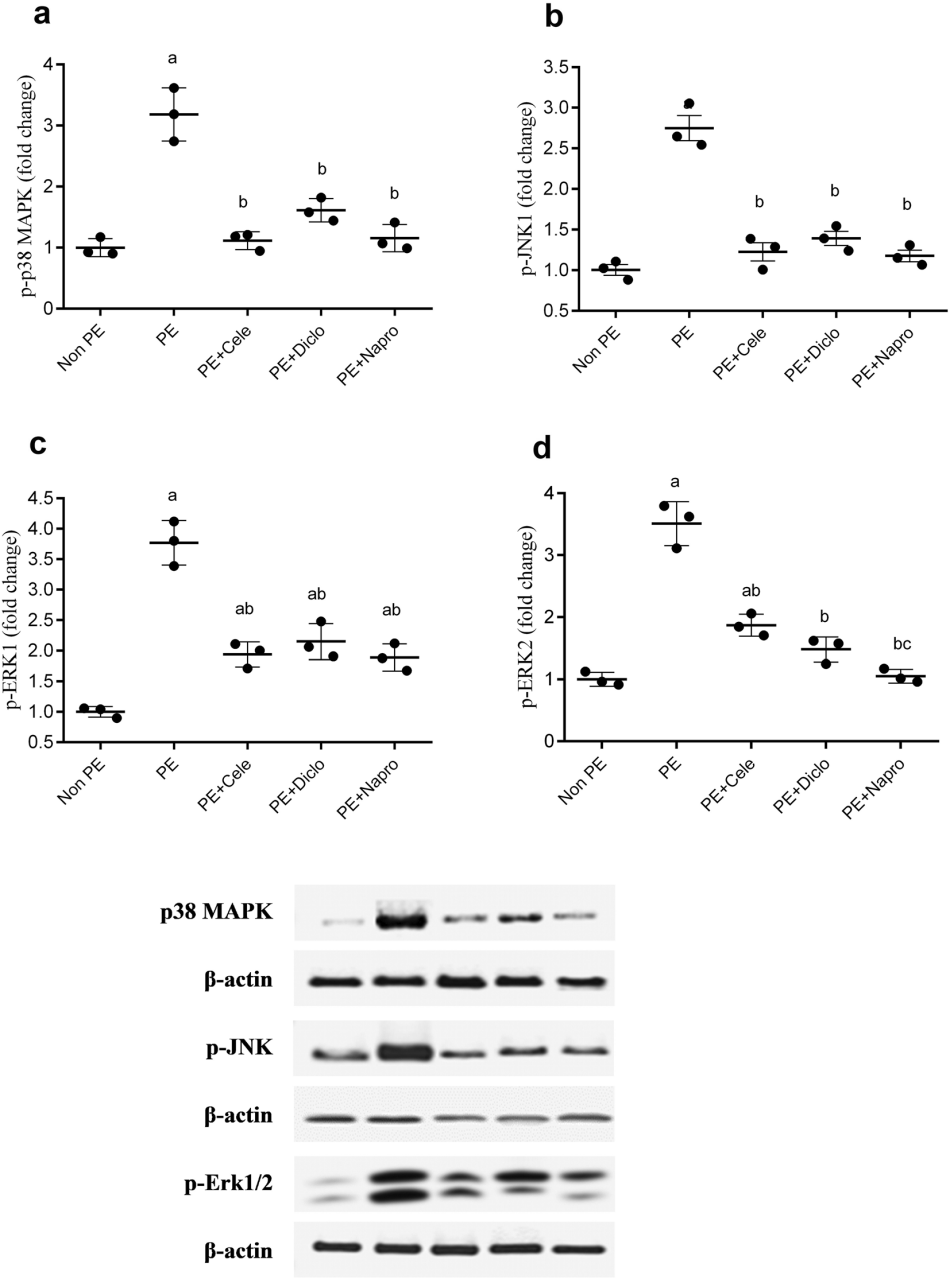
Effect of prenatal administration of celecoxib, diclofenac, or naproxen on renal protein expression of p-*p38 MAPK* (**a**), p-JNK (**b**), p-Erk1 (**c**) and p-Erk2 (**d**) in weaning PE rats. Values are means ± S.D. of 3 rats. *P < 0.05 vs. non-PE group (a), PE (b), PE + celecoxib (c), and PE + diclofenac (d) according to one-way-ANOVA (*F*-statistics, *p* > 0.0001).

Changes in renal contents of the cyclooxygenase products of arachidonic acid caused by PE in the absence and presence of NSAIDs are shown in Fig. 4. ELISA determinations showed significantly higher levels of PGE2, PGF2α and TXA2 in weaning PE dams compared with respective control values (Fig. 3). The prenatal treatment with NSAIDs significantly reduced the PE-evoked elevations in renal prostanoids, with an order of potency of naproxen > celecoxib > diclofenac (Fig. 3).

### Effects of gestational NSAIDs on renal MAPKs

Western blotting showed that the protein expressions of all MAPK isoforms, p38, p-JNK, p-Erk1, and p-ERK2, were significantly increased in kidneys of weaning PE dams. In fact, the renal expression of all MAPKs was at least tripled in PE compared with non-PE rats. Except for p-ERK2, the heightened expression MAPKs expression was mostly and indiscriminately eliminated by gestational administration of the 3 NSAIDs (Fig. 4). Notably, the PE-mediated increase in renal p-ERK2 was also suppressed by all NSAIDs, but the magnitude of this effect depended on the NSAID type. The efficacy order of p-ERK2 downregulation was naproxen > diclofenac > celecoxib (Fig. 4d).

### Effects of gestational NSAIDs on renal oxidative, fibrotic, and autophagic markers

**Figure 5:**
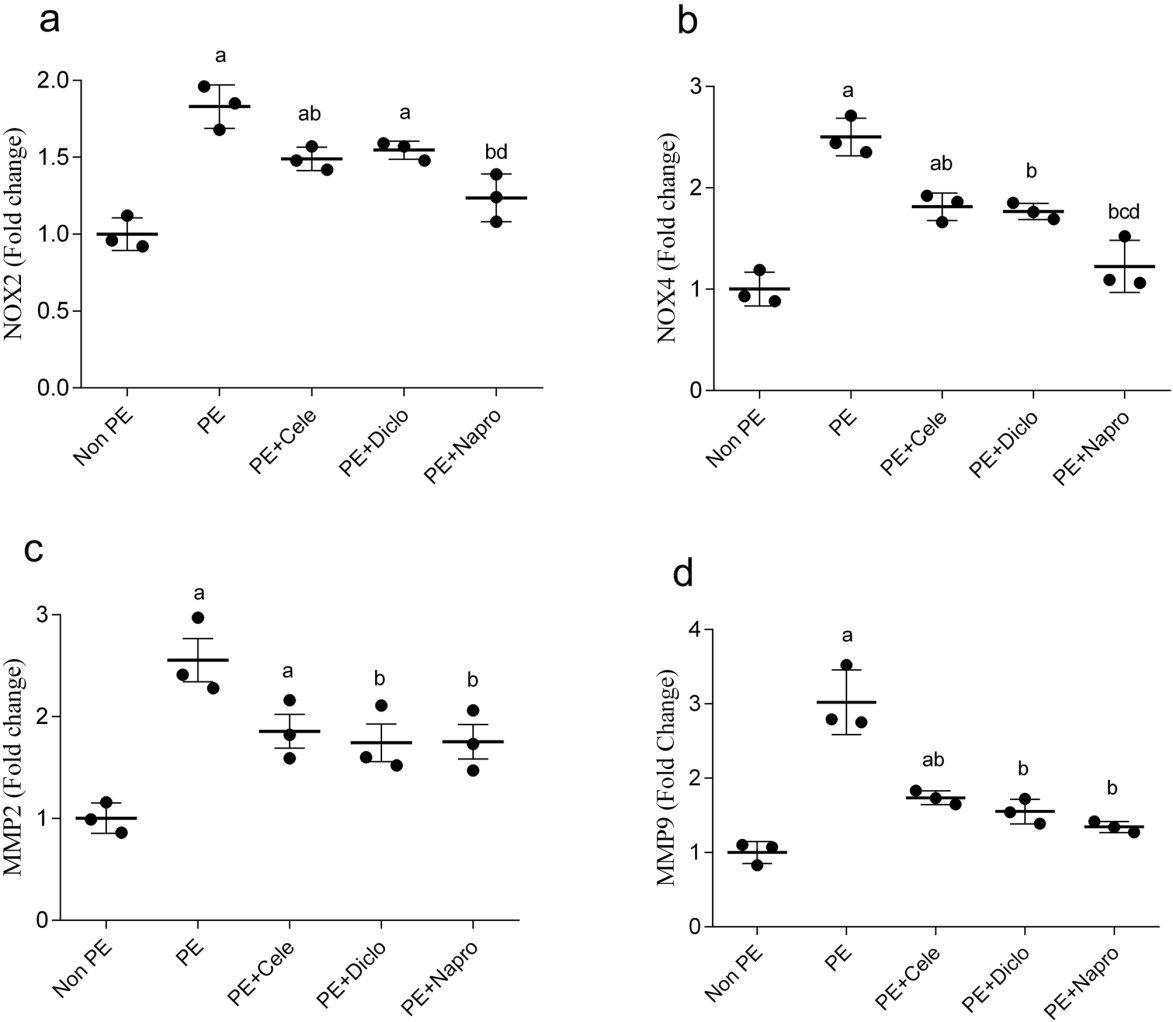
Effect of prenatal administration of celecoxib, diclofenac, or naproxen on renal gene expression of NOX2 (**a**), NOX4 (**b**), MMP2 (**c**) and MMP9 (**d**) in weaning PE rats. Values are means ± S.D. of 3 rats. *P < 0.05 vs. non-PE group (a), PE (b), PE + celecoxib (c), and PE + diclofenac (d) according to one-way-ANOVA (*F*-statistics, *p* = 0.0013–0.0001).

**Figure 6:**
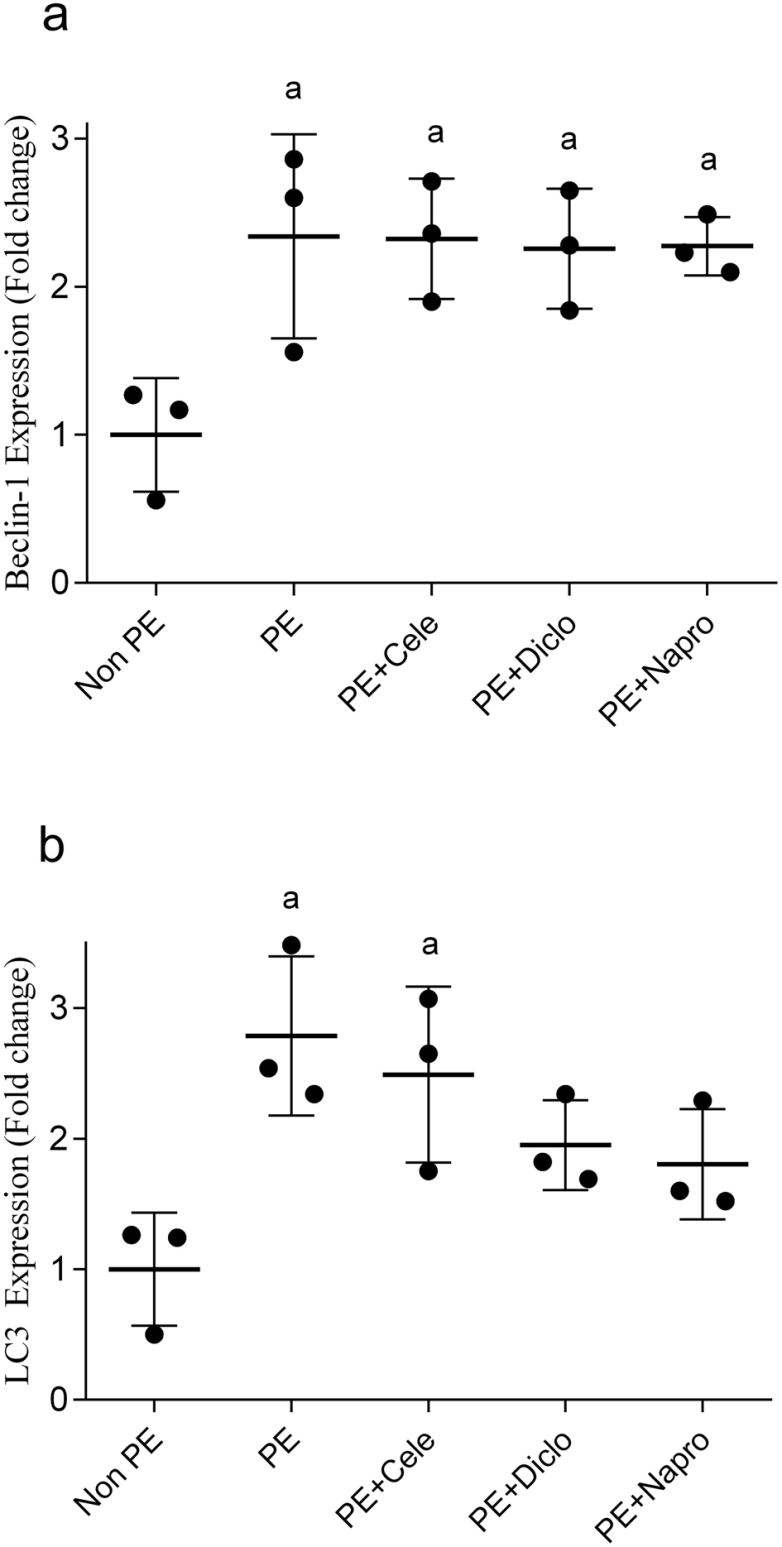
Effect of prenatal administration of celecoxib, diclofenac, or naproxen on renal gene expression of *beclin-1* (**a**) and LC3 (**b**) in weaning PE rats. Values are means ± S.D. of 3 rats. *P < 0.05 vs. non-PE group (a) according to one-way-ANOVA (*F*-statistics, *p* = 0.0165–0.0137).

PCR studies demonstrated significant increases in gene expressions of the NADPH oxidases, NOX2 and NOX4 (Fig. 5a, b), matrix metalloproteinases (MMP2 and MMP9, Fig. 5c, d), and autophagic markers (Beclin-1 and LC3, Fig. 6A, B) in renal tissues of weaning PE compared with non-PE rats. The deteriorated renal oxidative potential evidenced by the upregulated NOX isozymes was significantly relieved by all NSAID therapies, with naproxen being the most effective NSAID (Fig. 5a, b). On the other hand, while the PE-associated rises in renal expression of MMP2 were comparably reduced by all NSAIDs (Fig. 5c), the significant rise in renal MMP9 expression by PE disappeared in dams receiving diclofenac or naproxen (Fig. 5D). Finally, none of the tested NSAIDs affected the surge caused by PE in the renal expression of the autophagy marker Beclin-1 (Fig. 6a). However, the simultaneous elevation in renal LC3 expression in PE kidneys was significantly diminished by naproxen or diclofenac, but not celecoxib (Fig. 6b).

## Discussion

Despite the increased risk of perinatal renal injury in preeclamptic mothers^8,9^ and the routine use of NSAIDs during pregnancy for the control of pain and inflammatory disorders^28^, we are not aware of any study that has determined if renal programming incited by PE could be modified by gestationally administered NSAIDs. The current study establishes a comparative investigation of the effect of prenatal administration of 3 prominent NSAIDs on renal damage provoked by PE in weaning mothers. Compared with celecoxib or diclofenac, naproxen appeared to be more advantageous in relieving functional and morphological manifestations of renal injury. Molecularly, the suppression of the upregulated offending signals of inflammatory (arachidonate-derived prostanoids, p-ERK2), oxidative (NOX2/4), fibrotic (MMP9), and autophagic (LC3) cascades provides a mechanistic basis for the privileged action of naproxen.

Consistent with reported clinical and experimental studies of exacerbated renal damage and morbidity and mortality in PE dams^29,30^, the present study demonstrated distinct functional and structural manifestations of renal injury in the L-NAME rodent model of PE. Opposite changes in urine protein (increases) and CrCl (decreases), positive hallmarks of renal damage^31,32^, were demonstrated in PE dams at GD20 compared with respective control rats and remained manifest till weaning (3 weeks post-labor). The excessive proteinuria has been attributed to the L-NAME-induced renal vascular wall thickening and elevated glomerular pressure^33^. Others suggested that the L-NAME-induced glomerular hypertension creates sheer stress that facilitates the urinary loss of protein^34^. Additionally, like in previous reports^29^, the disturbed kidney function in PE rats was accompanied by a number of pathological lesions such endotheliosis, cloudy swelling of the tubular epithelial lining, necrosis and reduced glomerular area.

The principal objective of the current study was to assess if and how the deteriorated renal profile in PE dams would be influenced by gestational supplementation of NSAIDs. The data showed that the net renal effect of individual antiinflammatory therapies was proportional to the magnitude of suppression of prostanoid biosynthesis. In this context, naproxen was more effective than the other two NSAIDs (celecoxib and diclofenac) in (i) rectifying biochemical and glomerular and tubular signs of renal injury caused by PE programming, and (ii) diminishing the upregulated renal levels of the arachidonate end products PGE2, PGF2α and TXA2. It is widely recognized that TXA2 and PGF2α are vitally implicated in the preeclamptic inflammatory response and consequent tissue remodeling and stiffness^31,35^. Moreover, a pathogenic role for PGE2 has been reported in oxidative and inflammatory damage associated with PE Moreover, a pathogenic role for PGE2 has been reported in oxidative and inflammatory damage associated with PE^36,37^. The MAPK family modulates diverse cellular processes including inflammation, cell differentiation, cell growth, and cell death^38,39^. The phosphorylation of p38 and JNK is increased in human placental explants after exposure to various PE-associated stresses such as angiotensin II, hypoxia and inflammatory cytokines, suggesting a causal relationship between MAPKs and PE pathophysiology^40^. Moreover, the inflammatory response provoked by p38 and ERK remodels spiral artery, interrupts trophoblast invasion, and consequently PE-like symptoms induced by lipopolysaccharides^41,42^. Western blotting data of the current study revealed that the expressions of all MAPK isoforms (p-p38, p-JNK, and p-ERK1/2) were upregulated in renal tissues of PE dams and this effect subsided after antenatal administration of NSAIDs. Notably, the depression of MAPK expression caused by all 3 NSAIDs was of similar magnitude, with the only exception that the reduced availability of renal p-ERK2 was more evident with naproxen than with celecoxib or diclofenac. Although ERK1 and ERK2 are co-expressed and regulated similarly in virtually all tissues and phosphorylate common subset of substrates in the cytosol and nucleus, ERK2 appears to be the predominant isoform in brain and hematopoietic cells^43–45^. Recent evidence reveals dynamic differences in the function and interplay between ERK1 and ERK2 in the regulation of cell signaling and proliferation^46^. Together, our data specifically implicate the reduced abundance of ERK2 in the distinct counterbalancing action of gestational naproxen against renal complications induced by PE. More studies are necessary to further clarify the correlation between the two ERK isoforms in this interaction.

The greater inhibitory effect of naproxen on renal ERK2 expression was paralleled with more noticeable suppression of the PE-induced upregulation of the gene expression of MMP9 as well as NADPH oxidases of the NOX2 and NOX4. Remarkably, the excessive generation of reactive oxygen species in placental and renal tissues of PE dams is primarily incited by NADPH oxidases^47^. Further, MMPs are a family of zinc-dependent proteases that are extensively distributed in tissues and involved in the degradation and turnover of extracellular matrix components in normal and complicated pregnancies^48^. It is widely recognized that oxidative and fibrotic bursts are key constituents that fine-tune and transduce the inflammatory response prompted by MAPK signaling. Recent data from studies on cardiorenal injuries suggests that the activation of the pro-oxidant NADPH oxidase following MAPKs phosphorylation results in the upregulation of pro-fibrotic transforming growth factor β1 and extracellular degradation signals regulated by MMPs^49–51^. The latter molecules are necessary for provoking tissue transition from the inflammatory phase to the fibrotic remodeling phase^52,53^. Thus, it is likely that the downregulation of consecutive signals of the ERK2/NOX2/NOX4/MMP9 cascade is causally related to naproxen renoprotection against preeclamptic renal injury.

Autophagy is a conserved recycling process that regulates lysosomal degradation and removal of dysfunctional cellular organelles and invading pathogens and plays key roles in fertilization and embryonic development^54^. The question whether PE and associated inflammatory and oxidative insults are positively or negatively modulated by autophagy remains unresolved^55–57^. Here we report two novel observations regarding the role of autophagy in the renal PE/NSAID interaction. First, the gene expression of LC3 and beclin-1, two autophagy-related markers^58^, were potentiated in renal tissues of weaning PE dams, which is coherent with an offensive role for autophagy in the pathophysiological events leading to PE. The suppression of angiogenetic and vascular endothelial activities has often been incriminated in the disrupting effect of autophagy during PE^56^. We further show that while the elevated renal beclin-1 expression was altered by none of the NSAID therapies, the concomitant rise in renal LC3 expression was diminished by diclofenac and naproxen, but not celecoxib. The data emphasizes the importance of downregulation of the LC3-dependent autophagic pathway in the renoprotection brought in by gestational naproxen and diclofenac in PE dams.

The current observation that antiinflammatory therapies counterbalance the PE-associated renal damage appears to be at odds with previous clinical and experimental assessments that highlight a compromising effect for NSAIDs on renal function. Depending on the dose and duration of use, acute and chronic kidney damage and tubulointerstitial nephritis are common adverse effects for NSAIDs that limit their use in clinical practice^23^. The mechanism possibly relates to the NSAIDs-mediated inhibition of vasodilator prostanoids, e.g. PGE2 and prostacyclin, and consequent vasoconstriction and reduced renal blood flow^24^. That said, our current observation that NSAIDs restrain renal damage induced by PE would advocate for a conditioning effect for NSAIDs in the setting of PE. In a similar fashion, Talab et al. reported that the NSAID compound indomethacin potentiated the preconditioning effect of lithium against renal injury induced by ischemia/reperfusion injury^59^.

As the current study was performed in weaning preeclamptic rats, it is not clear whether NSAIDs would interact similarly with functional and morphometric renal consequences of PE when tested during early postpartum days. This is especially important as the PE-associated elevations in blood pressure and renal glomerular filtration rate and proteinuria peak immediately after delivery and begin to gradually decline over subsequent months^60^. More studies are also necessary to determine whether (i) the PE/NSAID interaction could be replicated with other experimental PE models such as the reduced uterine perfusion pressure model^61^, and (ii) gestational administration of NSAIDs could modulate PE-induced neonatal illnesses and reductions in neonatal number and weight is^62,63^. Finally, the electron microscopic visualization of podocytes and glomerular endothelial cells might provide important insights into the PE/NSAIDs renal interaction. The investigation of these issues provides a framework for future studies in our laboratory to assess the PE/NSAIDs interaction on disturbances in renal homeostasis and possibly other end organ damage.

In conclusion, the present functional, histopathological, and molecular evidence suggests a preferential favorable effect for naproxen when administered during the last week of pregnancy on PE programming of renal function in weaning dams. The diminution of PE-mediated inflammatory, oxidative, fibrotic, and autophagic offences underlies the renoprotective action of naproxen. More experimental and clinical studies are necessary to reinforce such renoprotective action of naproxen in PE and underlying cellular mechanisms.

## Materials and methods

### Materials

*N*(gamma)-nitro-l-arginine methyl ester** (l**-NAME; sigma-Aldrich, St. louis. MO, USA), naproxen (Sigma, St. Louis, MO, USA) and thiopental sodium (Biochemie GmbH, Vienna, Austria) were purchased from commercial suppliers. Celecoxib and diclofenac were supplied as gifts from PHARCO Corporation, Alexandria, Egypt and Amriya Pharmaceutical Industries, Alexandria, Egypt, respectively. All drugs were dissolved in saline. All ELISA kits were acquired from Chongqing Biospes Co. (Chongqing, China) or Abcam (Cambridge, UK). Kits for RNA isolation and reverse transcription were bought from Qiagen (USA). Western Blot technique using V3 Western Workfow™ Complete System was acquired from Bio-Rad^®^ Hercules, CA, USA.

### Experimental animals

The study was performed in the Animal House of Pharos University in Alexandria, Alexandria, Egypt. Thirty female Wistar rats weighing 170–200 g were kept at 23–25 °C in a 12/12 h light/dark cycle under optimum humidity with access to food and water ad libitum. The experiments were conducted in adherence to the guidelines of the Egyptian guide for the care and use of laboratory animals^64^ and in accordance with the Unit of Research Ethics Approval Committee, Pharos University in Alexandria, Egypt (Approval No.: PUA01202002233008) and with ARRIVE guidelines and the National Institutes of Health, USA, for the care and use of laboratory animals.

### PE induction

Adult nulliparous female rats were housed with male rats (ratio 1:1) and mated overnight. The date of conception was determined by checking sperms in the vaginal smears or detecting a vaginal plug. PE was induced by oral administration of l-NAME using oral gavage at a dose of 50 mg/kg/day for 7 continuous days from gestational day14 (GD14)^65–67^. Non-PE rats received water by gavage to correct for the stress of manipulation.

### Urine collection

Dams, at GD20 and weaning time, were housed in metabolic cages with stainless steel wire mesh bottom for 1 day prior to sacrifice, and 24-h urine samples were collected under light oil for measuring protein and creatinine. The collected urine was stored at −80 °C until processed.

### Experimental groups and study design

Pregnant female rats were randomly assigned into five different groups; n = 5–6 rats each: (i) pregnant non-PE control rats, (ii) PE rats, (iii) PE/celecoxib (10 mg/kg/day)^68^, (iv) PE/diclofenac (0.5 mg/kg/day)^69^, and (v) PE/naproxen (1 mg/kg/day)^70^. NSAIDs were co-administered with l-NAME starting at GD14 till GD20. At weaning (3 weeks post-labor), dams were euthanized by i.p. injection of an overdose of thiopental (100 mg/kg) and blood was collected via cardiac puncture, spun (800×g, 4 °C, 20 min), and serum was stored at −80 °C until used for the measurement of creatinine levels. Portions of the left kidneys were dissected, immediately frozen in liquid nitrogen, and stored at − 80 °C for protein and gene expression studies. Right kidneys were fixed in 10% formaldehyde and embedded in paraffin blocks for histopathological examination and morphometric analysis.

### Biochemical measurements

The levels of serum and urine creatinine were assessed using Jaffe' reaction^71^, then creatinine clearance (CrCl) was calculated. Urine protein level was measured using pyrogallol red method^72^. Total protein levels in kidney tissues were measured using the Lowry method^73^.

### Enzyme-linked immunosorbent assay (ELISA) determinations

The left kidney portions were homogenized in phosphate buffered saline in ratio of 1:9 and used to determine prostaglandin E2 (PGE2) and *prostaglandin F2 alpha (*PGF2α) (Enzo Life Sciences, NY, USA) and thromboxane A2 (TXA2) (LifeSpan BioSciences, Inc, Seattle, USA) using commercially available ELISA kits.

### Real time reverse transcriptase-polymerase chain reaction (qRT-PCR)

Total RNA was isolated from kidney tissue using the miRNeasy kit according to the manufacturer's instructions. The isolated RNA was reverse transcribed into complementary DNA (cDNA) using reverse transcriptase, amplified, and detected by qRT-PCR using specific primers. The primer sequences of the genes examined; beclin-1, microtubule-associated protein 1A/1B-light chain 3 (LC3), MMP2, MMP9, NADPH oxidase 2 (NOX2), NADPH oxidase 4 (NOX4) and the housekeeping gene glyceraldehyde-3-phosphate dehydrogenase (GAPDH) are shown in Table 1. Reverse transcription of all RNA species into cDNA was performed using the miScript II RT Kit (Qiagen, Germany) according to the manufacturer's instructions. The cDNA was used to quantify kidney expression of the investigated genes by Rotor-Gene Q qPCR using the QuantiTect SYBR Green PCR Master Mix. PCR amplification was initiated with an initial denaturation at 95 °C for 10 min and subsequent amplification by 40 PCR cycles as follows: Denaturation at 95 °C for 15 s, annealing at 58 °C for 15 s and extension at 60 °C for 15 s. Threshold cycle (Ct) values were determined using Rotor-Gene Q-Pure Detection version 2.1.0. (build 9). For each gene, changes in sample mRNA levels were determined using the 2^−ΔΔCt^ method and normalized to the reference gene^31^.

**Table 1:** Primers sequences of studied genes.

Gene name	Access no.	Sequence
Beclin-1	NM_001034117	F: TTGGCCAATAAGATGGGTCTGAA
		R: TGTCAGGGACTCCAGATACGAGTG
LC3	NM_022867	F: CAGGATCCATGCCGTCCCAGAAGACC
		R: GTCCCTTTTTGCCTTGGTAG
MMP2	NM_031054	F: ACCGTCGCCCATCATCAA
		R: TTGCACTGCCAACTCTTTGTCT
MMP9	NM_031055.2	F: TCGAAGGCGACCTCAAGTG
		R: TTCGGTGTAGCTTTGGATCCA
NOX2	NM_023965.1	F: TCTTTGTCATTCTGGTGTGGTTGG
		R: AGAGCCAGTGCTGACCCAA
NOX4	NM_053524.1	F: GGATCACAGAAGGTCCCTAGCA
		R: GCTACATGCACACCTGAGAAAATAC
GAPDH	NM_017008	F: TGCATCCTGCACCACCAACTGC
		R: ACAGCCTTGGCAGCACCAGTGG

### Western blotting

The ReadyPrepTM protein extraction kit (total protein) provided by Bio-Rad Inc (Catalog #163-2086) was employed according to manufacturer instructions was added to each sample of the homogenized tissues of all different groups. A Bradford assay was performed to determine protein concentration in each sample (Bio basic Inc., Markham Ontario, Canada). A 20 μg protein concentration of each sample was then loaded with an equal volume of 2× Laemmli sample buffer containing 4% SDS, 10% 2-mercaptoehtanol, 20% glycerol, 0.004% bromophenol blue and 0.125 M Tris HCl. The pH was checked and brought to 6.8. Each mixture was boiled at 95 °C for 5 min to ensure denaturation of protein before loading on polyacrylamide gel electrophoresis. Polyacrylamide gels were performed using TGX Stain-Free™ FastCast™ Acrylamide Kit (SDS-PAGE), which was provided by Bio-Rad Laboratories Inc (Cat # 161-0181). The gel was assembled in transfer sandwich as following from below to above (filter paper, PVDF membrane, gel and filter paper). The sandwich was placed in the transfer tank with 1× transfer buffer, which was composed of 25 mM Tris and 190 mM glycine and 20% methanol. Then, the blot was run for 7 min at 25 V to allow protein bands transfer from gel to membrane using BioRad Trans-Blot Turbo. The membrane was blocked in tris-buffered saline with Tween 20 (TBST) buffer and 3% bovine serum albumin at room temperature for 1 h. The components of blocking buffer were as follow; 20 mM Tris pH 7.5, 150 mM NaCl, 0.1% Tween 20 and 3% bovine serum albumin. Primary antibodies of phospho-p38 mitogen-activated protein kinases (p-p38 MAPK), *phospho-c-Jun N-terminal kinase* (p-JNK), extracellular signal-regulated protein kinase (ERK) p-Erk1, and p-ERK2 were diluted in TBST according to manufactured instructions. Incubation was done overnight in each primary antibody solution, against the blotted target protein at 4 °C. Blots were cut prior to hybridization with antibodies during blotting. The blot was rinsed 3–5 times for 5 min with TBST. Incubation was done in the HRP-conjugated secondary antibody (Goat anti-rabbit IgG- HRP-1mg Goat mab -Novus Biologicals) solution against the blotted target protein for 1 h at room temperature. The chemiluminescent substrate (Clarity TM Western ECL substrate Bio-Rad cat#170-5060) was applied to the blot according to the manufacturer’s recommendation. Briefly, equal volumes were added from solution A (Clarity western luminal/enhancer solution) and solution B (peroxidase solution). The chemiluminescent signals were captured using a CCD camera-based imager. The densities of phosphorylated protein bands were expressed as ratios of respective β-actin bands. Image analysis software was used to read the band intensity of the target proteins against control sample beta actin (housekeeping protein) by protein normalization on the ChemiDoc MP imager^74,75^.

### Histological and histomorphometric analysis

Kidneys were fixed in 10% neutral buffered formalin for 48 h, embedded in paraffin, and slices of 4–5 μm were stained with hematoxylin & eosin (H&E) and examined for structural defects at  200× and  400×. Morphometric determinations of the glomerular count and mean area percentage were performed in 20 random fields per rat and averaged. Areas of the glomerular capillary tufts were circumferenced on images captured at  200×. The ImageJ software (1.52p software 32, NIH, USA) was employed to measure the circumferenced areas using the fit ellipse parameter and presented as a percentage to the whole image^76,77^.

### Statistical analysis

The findings are presented as mean ± standard deviation (SD) and analyzed using one-way or two-way ANOVA and *F*-statistics as appropriate, followed by Tukey post-hoc test with a probability level (P) ≤ 0.05 taken as the limit for significance. The GraphPad Prism v7.0 (GraphPad Prism Inc., La Jolla, CA, USA) was utilized for these analyses (Supplementary Information).

### Supplementary Information

Supplementary Information.

## Data Availability

Raw data are publicly available in the Mendeley repository, as part of this record: https://data.mendeley.com/datasets/hg7h7883gz/1^78^.
