# Supplementary material for: The suppression of MAPK/NOX/MMP signaling prompts renoprotection conferred by prenatal naproxen in weaning preeclamptic rats
Source: Sci Rep. 2023 Oct 15;13:17498. doi: 10.1038/s41598-023-44617-2 (PMC10577149; doi:10.1038/s41598-023-44617-2)
Supplement: Supplementary file 1 — Supplementary Information. [file 41598_2023_44617_MOESM1_ESM.pdf]

**p38 MAPK**	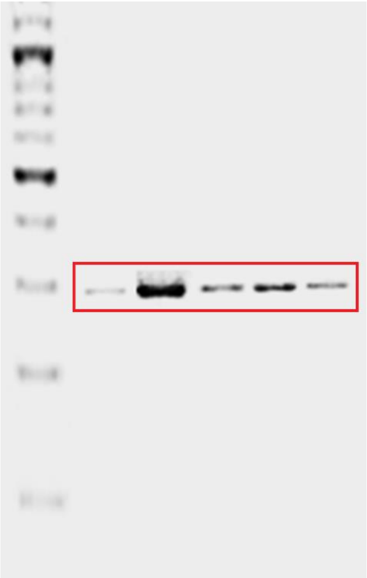	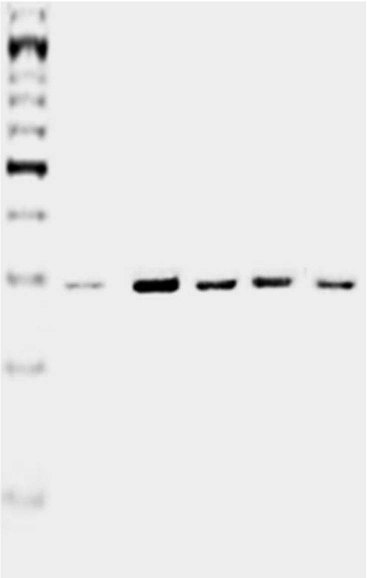	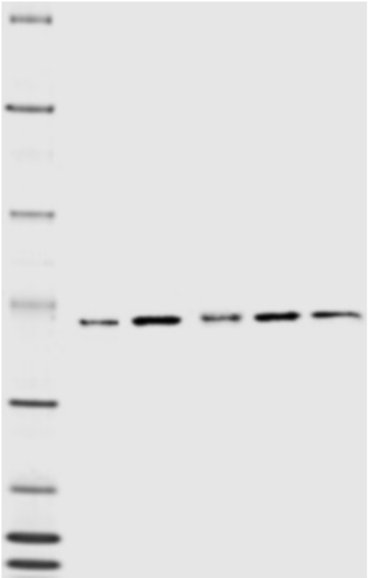	38 kDa
**β-actin**	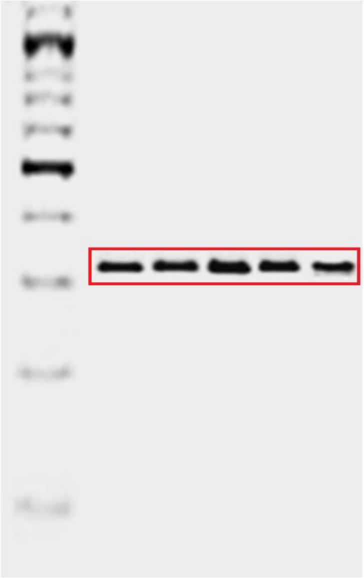			43 kDa

**p-JNK**	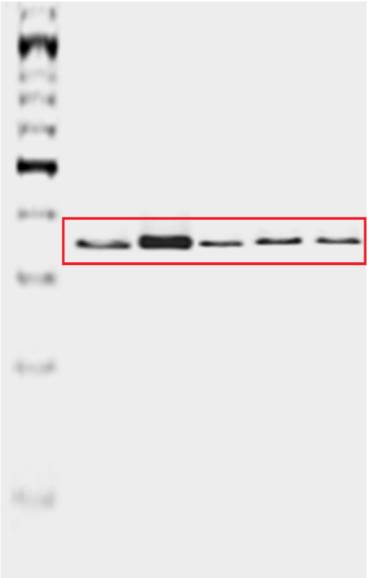	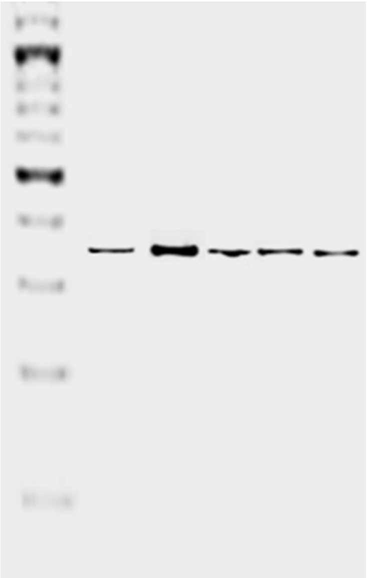	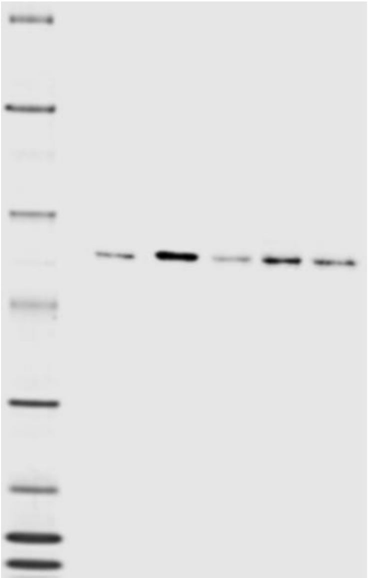	54 kDa
**β-actin**		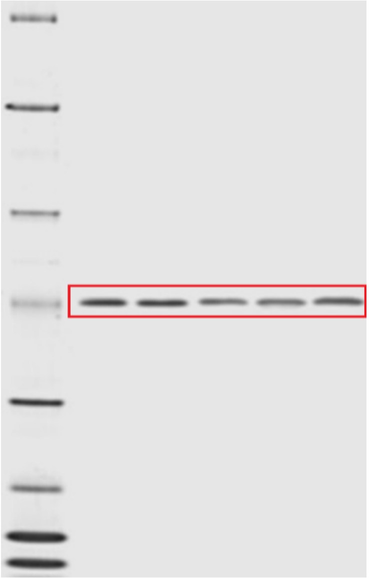		43 kDa

**p-Erk1/2**	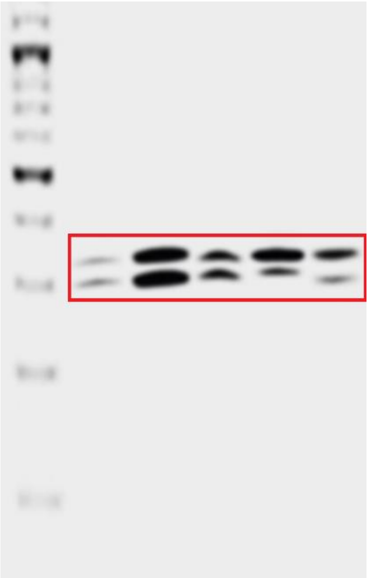	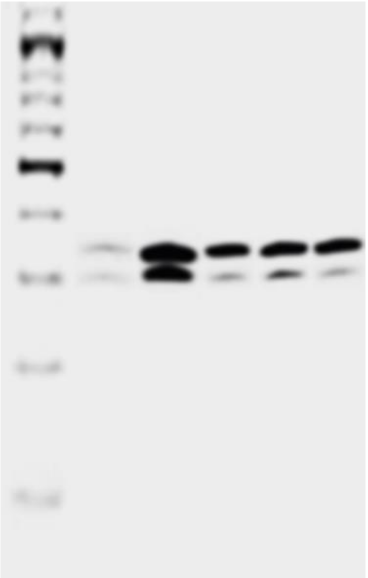	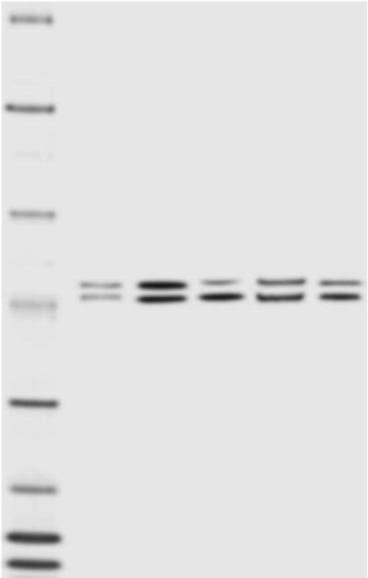	44 kDa<br>42 kDa
**β-actin**		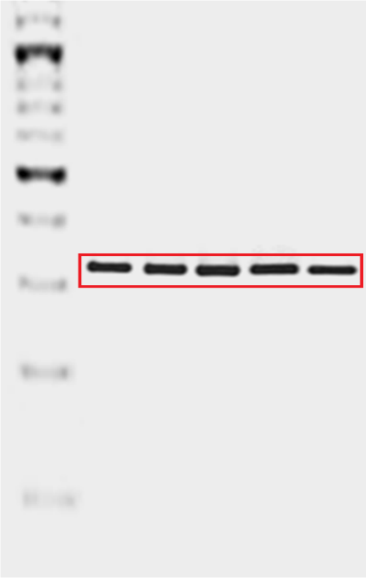		43 kDa

**Blot 1**	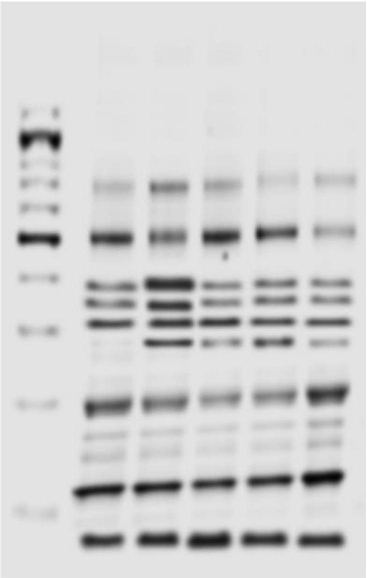
**Blot 2**	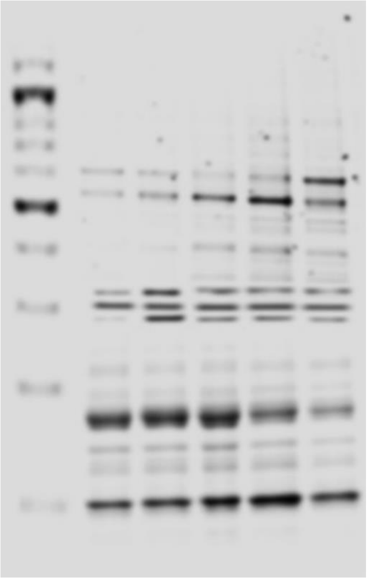

**Blot 3**	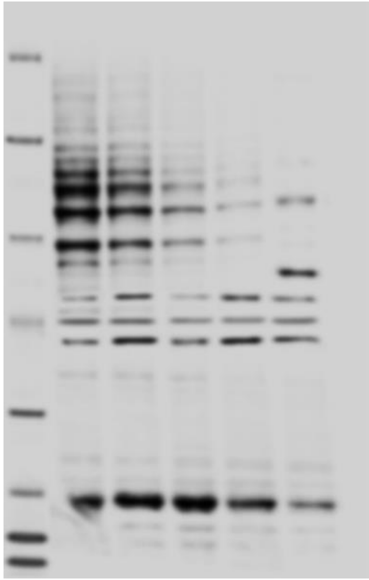
